# Sudden unexpected death in asymptomatic infants due to *PPA2* variants

**DOI:** 10.1002/mgg3.1008

**Published:** 2019-11-09

**Authors:** Colin K. L. Phoon, Matthew Halvorsen, David B. Goldstein, Rachel Rabin, Frank Cecchin, Laura Crandall, Orrin Devinsky

**Affiliations:** ^1^ Division of Pediatric Cardiology New York University School of Medicine Hassenfeld Children’s Hospital at NYU Langone New York NY USA; ^2^ Department of Pediatrics New York University School of Medicine Hassenfeld Children’s Hospital at NYU Langone New York NY USA; ^3^ Department of Genetics University of North Carolina Chapel Hill NC USA; ^4^ Institute for Genomic Medicine Columbia University Medical Center New York NY USA; ^5^ Department of Neurology New York University School of Medicine New York NY USA

**Keywords:** cardiomyopathy, mitochondrial disease, myocarditis, PPA2, sudden death

## Abstract

**Background:**

Sudden death in children is a tragic event that often remains unexplained after comprehensive investigation. We report two asymptomatic siblings who died unexpectedly at approximately 1 year of age found to have biallelic (compound heterozygous) variants in *PPA2*.

**Methods:**

The index case, parents, and sister were enrolled in the Sudden Unexplained Death in Childhood Registry and Research Collaborative, which included next‐generation genetic screening. Prior published cases of *PPA2* variants, along with the known biology of *PPA2*, were also summarized.

**Results:**

Whole exome sequencing in both siblings revealed biallelic rare missense variants in *PPA2*: c.182C > T (p.Ser61Phe) and c.380G > T (p.Arg127Leu). *PPA2* encodes a mitochondrially located inorganic pyrophosphatase implicated in progressive and lethal cardiomyopathies. As a regulator and supplier of inorganic phosphate, PPA2 is central to phosphate metabolism. Biological roles include the following: mtDNA maintenance; oxidative phosphorylation and generation of ATP; reactive oxygen species homeostasis; mitochondrial membrane potential regulation; and possibly, retrograde signaling between mitochondria and nucleus.

**Conclusions:**

Two healthy and asymptomatic sisters died unexpectedly at ages 12 and 10 months, and were diagnosed by molecular autopsy to carry biallelic variants in *PPA2*. Our cases add additional details to those reported thus far, and broaden the spectrum of clinical and molecular features of *PPA2* variants.

## Graphical abstract

Two healthy and asymptomatic sisters died unexpectedly at ages 12 and 10 months, and were diagnosed by molecular autopsy to carry biallelic variants in PPA2. This gene encodes a mitochondrially located inorganic pyrophosphatase implicated in progressive and lethal cardiomyopathies.

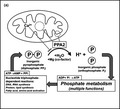

## 1 INTRODUCTION

Sudden death in childhood and infancy is a tragic event that often remains unexplained after comprehensive investigation. Newer, registry‐based investigations such as the SUDC (Sudden Unexplained Death in Childhood) Registry and Research Collaborative have led to molecular insights into many of these premature deaths. Here, we report two asymptomatic siblings who died unexpectedly at approximately 1 year of age, with biallelic (i.e., compound heterozygous) variants in *PPA2* (OMIM 609988; GenBank Reference Sequence NG_053007.1). We review and discuss the published data on *PPA2* biallelic variants.

## 2 CASE HISTORIES

The index case was a 12‐month‐old Caucasian girl who was healthy and asymptomatic, with normal growth and development until 2 days before death. The prenatal history was unremarkable; mother smoked 10 cigarettes per day during pregnancy. The infant's only diagnosis was “bronchitis” at age 6 months. In the 2 days before death, she experienced fever, vomiting, diarrhea, and poor appetite. The day before her death, she was sleepy and was not especially active; she fell over a few times and looked like “her legs gave out.” Throughout her last night alive, she had episodes of vomiting and diarrhea. During the last episode, she vomited twice and then seemed to gasp for air; mother felt her breathing change, her eyes rolled backward, and she stopped breathing. At autopsy, there were nonspecific features, which together with circumstances of a witnessed collapse, suggested a cardiac arrhythmia including: (a) contraction bands in cardiac muscle fibers, which while not pathological, suggested an acute cardiac event; (b) pleural and pericardial effusions, and ascites which may be secondary to resuscitation, but were considered more than is usually encountered; (c) prominent intra‐alveolar hemorrhage and acute pulmonary edema which could be secondary to resuscitation, but was considered more striking than usual.

Family history revealed a sister who had died suddenly at 10 months of age, a death that was attributed to a cardiac arrhythmia (autopsy report noted that she was witnessed to have screamed, gasped for breath, and became rigid and blue) but whose death was certified as a sudden infant death syndrome (SIDS) case. This sibling had research genetic testing through the National Health Service: 169 genes were sequenced (38 genes associated with arrhythmia, with no pathogenic variants found). The mother had a history of episodic syncope of unknown etiology, not provoked by exercise or other factors, beginning at 13 years of age through her first pregnancy at 18 years of age.

The index case, parents, and sister were then enrolled in the SUDC Registry and Research Collaborative (SUDCRRC). To enroll in the SUDCRRC, parents consented to release deidentified data for research. The Foundation provided deidentified data for analysis. The New York University Institutional Review Board considered this study exempt. Testing included genetic screening at the Institute for Genomic Medicine (IGM) at Columbia University. Whole exome sequencing in both siblings revealed biallelic rare missense variants in *PPA2*: c.182C > T (p.Ser61Phe) and c.380G > T (p.Arg127Leu). PolyPhen‐2 predicted these compound heterozygous variants to be “probably damaging”: p.Ser61Phe score, 0.998 (sensitivity, 0.27; specificity, 0.99); and p.Arg127Leu score, 0.993 (sensitivity, 0.70; specificity, 0.97) (http://genetics.bwh.harvard.edu/pph2/, accessed 05/11/2019; Adzhubei et al., 2010). Neither variant is observed in the homozygous state in population databases, such as gnomAD (Lek et al., 2016). Prior cases of *PPA2* variants are shown in the Table 1, in addition to our cases.

**Table 1:** Clinical histories

Ref.	Patient	M/F	Variant 1	Variant 2	Genetics	Symptoms	Premortem cardirac diagnosis (including arrhythmia)	Age at death	Postmortem findings
Case	F1:P1	F	c.182C > T	c.380G > T	Biallelic, compound het	Fever, vomiting, and diarrhea, and poor appetite in 2 days prior to death	None	12 m	Contraction bands in cardiac muscle fibers; pleural and pericardial effusions, ascites; prominent intra‐alveolar hemorrhage and pulmonary edema
Case	F1:P2	F	c.182C > T	c.380G > T	Biallelic, compound het	None	None	10 m	Details not available: Diagnosed as SIDS case, attributed to possible cardiac arrhythmia
G16	F1:P1	M	N/A	N/A	N/A	Unable to walk unaided at 14 months; fever 3 weeks prior to death, with pallor in intervening period	None	14 m	N/A
G16	F1:P2	F	c.182C > T	c.881A > C	Biallelic, compound het	At 15 months, +chicken pox; at 16 months, +viral illness with fatigue; 1 week later, pale ‐ hospitalized the next day for suspicion of myocarditis. Died in the ICU 6 hr later of cardiac arrest	None	14 m	Myocarditis^a^
G16	F1:P3	F	c.182C > T	c.881A > C	Biallelic, compound het	Mother and maternal uncle with + Brugada syndrome, but infant monitoring was normal. +Otitis media, 5 weeks prior to death. Pale on the day of hospitalization, with sinus tachycardia; then developed acute and severe bradycardia with arrest.	None	15 m	Massive circumferential fibrosis of myocardium, moderate inflammatory infiltrate^a^
G16	F1:P4	M	c.182C > T	c.881A > C	Biallelic, compound het	Mother and maternal uncle with Brugada syndrome. Good health with normal cardiac monitoring. ECG at 12 months normal, no changes to flecainide challenge. Sick and pale the day after the last echo; then + cardiac arrest at home.	None	14 m	Focal and discrete lesions of myocardic fibers and inflammatory infiltrate on the LV posterior wall with no fibrosis^a^
G16	F2:P1	F	c.280A > G	c.514G > A	Biallelic, compound het	+Pyelonephritis treated at 6 months. At 20 months, presented to clinic with poor appetite, vomiting, and pallor; gastroenteritis diagnosed and sent home. Cardiac arrest that night.	None	20 m	Focal myocarditis with moderate inflammatory infiltrate, some necrosis in LV; lesions of different ages^a^
G16	F2:P2	F	c.280A > G	c.514G > A	Biallelic, compound het	+Rhinopharyngitis 5 days prior to death, with poor appetite, pale and hypotonic; within a few hours, hospitalized for cardiac failure and died the same day during transport to the ICU.	None	4 m	Inflammatory infiltrate of pericardium, myocardium, and endocardium; lipid accumulation; and necrosis of myocytes^a^
G16	F3:P1	F	c.318G > T	c.514G > A	Biallelic, compound het	Myopathy‐Hypotonia, feeding difficulties, failure to thrive at 3 months of age; hypertrophic cardiomyopathy, with heart failure at 4 months. Died of cardiac arrest.	Hypertrophic cardiomyopathy with heart failure diagnosed at 4 months; no arrhythmias	4 m	Hypertrophic cardiomyopathy, +lipid accumulation^a^
K16	F1:P1	M	c.514G > A	c.683C > T	Biallelic, compound het	Healthy until age 15 years ‐ collapsed and died after ingestion of a small volume of beer: Had previously developed pallor and severe chest and arm pain after consumption of very small amounts of alcohol.	None	15 y	Both ventricles slightly dilated; small pale area in the epicardium of the left ventricle, evidence of focal inflammation. Diagnosis: myocarditis and sudden arrhythmic cardiac death.
K16	F1:P2	M	c.514G > A	c.683C > T	Biallelic, compound het	Sibling of F1:P4, with family history of sudden death and exquisite sensitivity to alcohol. Cardiac MRI showed marked mid‐myocardial fibrosis. Subsequently received an implantable defibrillator, although no events have occurred to date.	Cardiac MRI showed marked mid‐myocardial fibrosis	N/A	N/A
K16	F1:P3	M	c.514G > A	c.683C > T	Biallelic, compound het	Died suddenly at 20 years of age after ingestion of a small amount of alcohol (approx. 10 g ethanol)	None	20 y	Heart weighed 395 g (normal 300 g). Left ventricle dilated with a circumferential lamina of scarring in mid‐myocardium. Microscopic examination: Very widespread, mostly mature scarring of mid‐myocardium.
K16	F1:P4	M	c.514G > A	c.683C > T	Biallelic, compound het	Sibling of F1:P2, with family history of sudden death and exquisite sensitivity to alcohol. Cardiac MRI showed marked mid‐myocardial fibrosis. Subsequently received an implantable defibrillator, although no events have occurred to date.	Cardiac MRI showed marked mid‐myocardial fibrosis	N/A	N/A
K16	F2:P1	M	c.500C > T	c.500C > T	Biallelic, homozygous	Healthy until 10 days, when developed vomiting and loose stools. +Seizures, lactic acidosis, hypotonia on 11th day of life. Heart and lung function normal initially. ECG: +ST elevations. Died from cardiac arrest with severe bradycardia^b^.	Heart failure, arrhythmias	10 d	Herds of fresh necrosis mainly of the right heart and interstitial lymphocyte infiltration. Electron microscopy: Myocardium showed mitochondria with degeneration of cristae. No evidence of viral infection.
K16	F2:P2	F	c.500C > T	c.500C > T	Biallelic, homozygous	Well until 14 days of age, then sudden deterioration with marked tachypnea, vomiting, and seizures; also lactic acidosis, hypotonia, and cardiac arrhythmia. Died from acute cardio‐respiratory decompensation, 6 hr after admission^b^.	Heart failure, arrhythmias	14 d	Acute and subacute necrosis more pronounced in the right heart more severe than in the left heart. Electron microscopy: Myocardium showed mitochondria with degeneration of cristae similar to F2:P1
K16	F2:P3	M	c.500C > T	c.500C > T	Biallelic, homozygous	Observed in ICU from birth, well for first 2 days. Developed sweating, vomiting, fatigue, and elevations of lactate, CK, CK‐MB. +Heart failure with occasional arrhythmia that worsened. Died from intractable ventricular arrhythmias^b^.	Heart failure, arrhythmias	32 d	Myocardium with no necrosis or inflammatory infiltrations. Reduced amount of myofibrils in myocytes. Herd of fibrosis in right heart.
K16	F3:P1	F	c.500C > T	c.500C > T	Biallelic, homozygous	Well until 5.5 months of age. Admitted after 24‐hr history of vomiting and diarrhea; +seizures. Echo: poor cardiac contractility. Multiple cardiac arrests during transport to children's hospital, died^b^.	Heart failure, arrhythmias	5.5 m	+Rotavirus in stool, but tests for myocarditis negative. +Longstanding myocyte loss, increased interstitial collagen, focal myocyte fiber disarray in the LV and septum; did not meet criteria for hypertrophic cardiomyopathy.
K16	F3:P2		c.500C > T	c.500C > T	Biallelic, homozygous	Well until age 8 months, then + viral illness led to increasing hypotonia and weakness. +Lactic acidosis with elevated CK; echo normal. Improved, but then + vomiting and diarrhea (+Norovirus), seizures, and drowsiness at 11 months. Died from sudden cardiac arrest^b^.	Heart failure, arrhythmias	11 m	Extensive fibrosis of the heart muscle
K16	F4:P1	M	c.380G > T	c.514G > A	Biallelic, compound het	+Seizures at 10 months; developed dilated cardiomyopathy and multiorgan failure at 1 year. Echo at 14.5 months and 18 months showed normal LV function with mild hypertrophy. At 2 years, admitted with Norovirus gastroenteritis. Rapid deterioration, died from asystolic cardiorespiratory arrest^b^.	Heart failure, arrhythmias	2 y	Extensive transmural fibrosis of the left ventricle, acute myocardial ischemia due to mitochondrial myopathy; mild left ventricular hypertrophy
V18	F1:P1	M	c.514G > A	c.556G > A	Biallelic, compound het	Mild viral infection symptoms (diarrhea, vomiting) prior to the rapid deterioration of his condition	Rapidly progressive dilated cardiomyopathy and cardiac failure diagnosed only a few days from disease onset to death	8 m	Dilation of the left ventricle, evidence of focal fibrosis, and inflammatory infiltrates with acute myocyte loss
V18	F1:P2	M	c.514G > A	c.556G > A	Biallelic, compound het	Mild viral infection symptoms (diarrhea, vomiting) prior to rapid deterioration. Serial echo monitoring normal until the sudden disease manifestation, when both dilated cardiomyopathy and poor cardiac function developed.	Rapidly progressive dilated cardiomyopathy and cardiac failure diagnosed only a few days from disease onset to death	5 m	Dilation of the left ventricle, evidence of focal fibrosis, and inflammatory infiltrates with acute myocyte loss

Note

Patients are identified/grouped according to the family (“F”, family; “P”, patient); ages at death are abbreviated as “d” (days), “m” (months), and “y” (years). References: “G16” (Guimier et al., 2016); “K16” (Kennedy et al., 2016); “V18” (Vasilescu et al., 2018). Abbreviations: “CK”, creatine kinase; “ECG”, electrocardiogram; “het”, heterozygous; “ICU”, intensive care unit; “LV”, left ventricle or left ventricular. *PPA2*: GenBank Reference Sequence NG_053007.1.

a Bacterial and viral screening and testing for fatty acid oxidation deficiency in fibroblasts and/or by acylcarnitine profiling failed to provide a diagnosis (Guimier et al., 2016).

b Individuals from families 2, 3, and 4 in Kennedy et al.’s series (2016) exhibited classical mitochondrial disease symptoms and died within the first 2 years of life of cardiac failure: lactic acidosis, seizures, hypotonia, and cardiac arrhythmia within the first months of life. Died from cardiac failure after sudden deterioration: Interestingly, both individuals from family 3 and the affected individual from family 4 had viral infections at the time of hospital admission before their final heart failure.

## 3 DISCUSSION

We identified two sisters with biallelic variants in *PPA2*, which encodes a mitochondrially located inorganic pyrophosphatase (PPase) that is implicated in progressive and lethal cardiomyopathies. The combination of both the p.Ser61Phe and p.Arg127Leu variants in our compound heterozygotes has not been reported before; each of these variants was identified separately in two sudden death cases from two earlier case series (Guimier et al., 2016; Kennedy et al., 2016). Our cases represent the severe range of a clinical spectrum that exhibits sudden death in infancy with minimal prodromic symptoms, and further underscore the importance of *PPA2* variants in sudden, unexpected death and in cardiac biology.

Initially reported in yeast, *PPA2* (MIM: 609988, GenBank: NM_176869.2) codes for an inorganic PPase, localized to the mitochondrial matrix; the protein product of its counterpart *PPA1* localizes to the cytosol (Lundin, Baltscheffsky, & Ronne, 1991; Lundin, Deopujari, Lichko, Silva, & Baltscheffsky, 1992). Alternate transcriptional splice variants, encoding different isoforms, have been characterized. The protein encoded by *PPA2* is highly similar to other members of the inorganic pyrophosphatase family, and contains the signature sequence essential for PPase catalytic activity. PPases catalyze the hydrolysis of pyrophosphate to inorganic phosphate, essential for cellular phosphate metabolism and required in many processes including the synthesis of DNA, RNA, proteins, polysaccharides, and lipids, as well as energy metabolism (Guimier et al., 2016). *PPA2* is highly conserved across species (https://www.ncbi.nlm.nih.gov/gene/27068, accessed 12/25/18).

Because *PPA2* functions within the mitochondrial matrix, variants in this nuclear‐encoded protein cause mitochondrial diseases. In 2016, two separate case series described biallelic *PPA2* variants in several pedigrees exhibiting sudden death (Guimier et al., 2016; Kennedy et al., 2016). Moreover, a large Finnish cardiomyopathy registry of severe childhood cardiomyopathy (KidCMP) recently included two cases (index case + brother, out of 66) with a *PPA2* biallelic variant who died at 8 and 5 months of age, respectively. Given that the incidence of “severe” childhood cardiomyopathy is ~0.50 per 100,000 population under the age of 10–15 years, the estimated the incidence of cardiomyopathy due to *PPA2* biallelic variants would be approximately 0.01 per 100,000 (1 per 10,000,000) pediatric population (Vasilescu et al., 2018). Another estimate of the cumulative heterozygous carrier frequency of likely pathogenic *PPA2* variants is 0.0024; that is prevalence for compound heterozygous or homozygous pathogenic *PPA2* mutations of 0.58 per 100,000 (1 in 170,000; Kennedy et al., 2016). Notably, while most variants associated with sudden death in childhood are inherited in autosomal dominant fashion, *PPA2* variants exhibit an autosomal recessive pattern. There is therefore a 25% recurrence risk for families.

Many of the infants reported in these three series demonstrated a viral prodrome (often, a viral gastroenteritis) with pallor within days of their deaths. Our two siblings were asymptomatic except for a viral illness shortly before death in one; their deaths were sudden and unexpected. The initial genetic panel screening did not show any definite or likely pathogenic variants, although several variants of unknown clinical significance were identified. With the advent of next‐generation sequencing, molecular autopsies are now shedding insights into causes of sudden, unexpected death in infancy and childhood. Most causative variants, including our patients, occurred as biallelic variants or compound heterozygotes; only in one family (#2 in reference) was a homozygous variant (c.500C > T; p.Pro167Leu) reported. Neither of the individual variants in our cases (p.Ser61Phe; p.Arg127Leu) have been found in homozygous form in gnomAD (accessed 03/01/2019).

Biallelic *PPA2* variants display a spectrum of severity (Table 1). Personal histories have included seizures and lactic acidosis typical of mitochondrial diseases, and cardiac arrhythmia. Provocative factors associated with sudden death have included acute viral gastroenteritis in young children, and small amounts of alcohol (Family 1, Kennedy et al., 2016). Age of death has ranged from early infancy to adolescence. Autopsy findings have frequently demonstrated myocarditis, cardiac inflammation, and cardiac fibrosis, which our cases did not demonstrably exhibit.

None of the cases previously reported (Guimier et al., 2016; Kennedy et al., 2016; Vasilescu et al., 2018) harbored both the p.Ser61Phe and p.Arg127Leu variants. The p.Ser61Phe variant (associated with the additional allelic variant p.Gln294Pro, in family F1; Guimier et al., 2016) was associated with sudden death in early toddler age (14–15 months) and histological evidence of myocarditis. Kennedy et al. (2016) described a toddler (in family F4) carrying p.[Arg127Leu];[Glu172Lys] biallelic variants in whom mild left ventricular hypertrophy was diagnosed at age 10 months, associated with seizures; death occurred at 2 years of age, with histological evidence of extensive left ventricular fibrosis and acute myocardial ischemia.

### 3.1 Biology of PPA2

**Figure 1:**
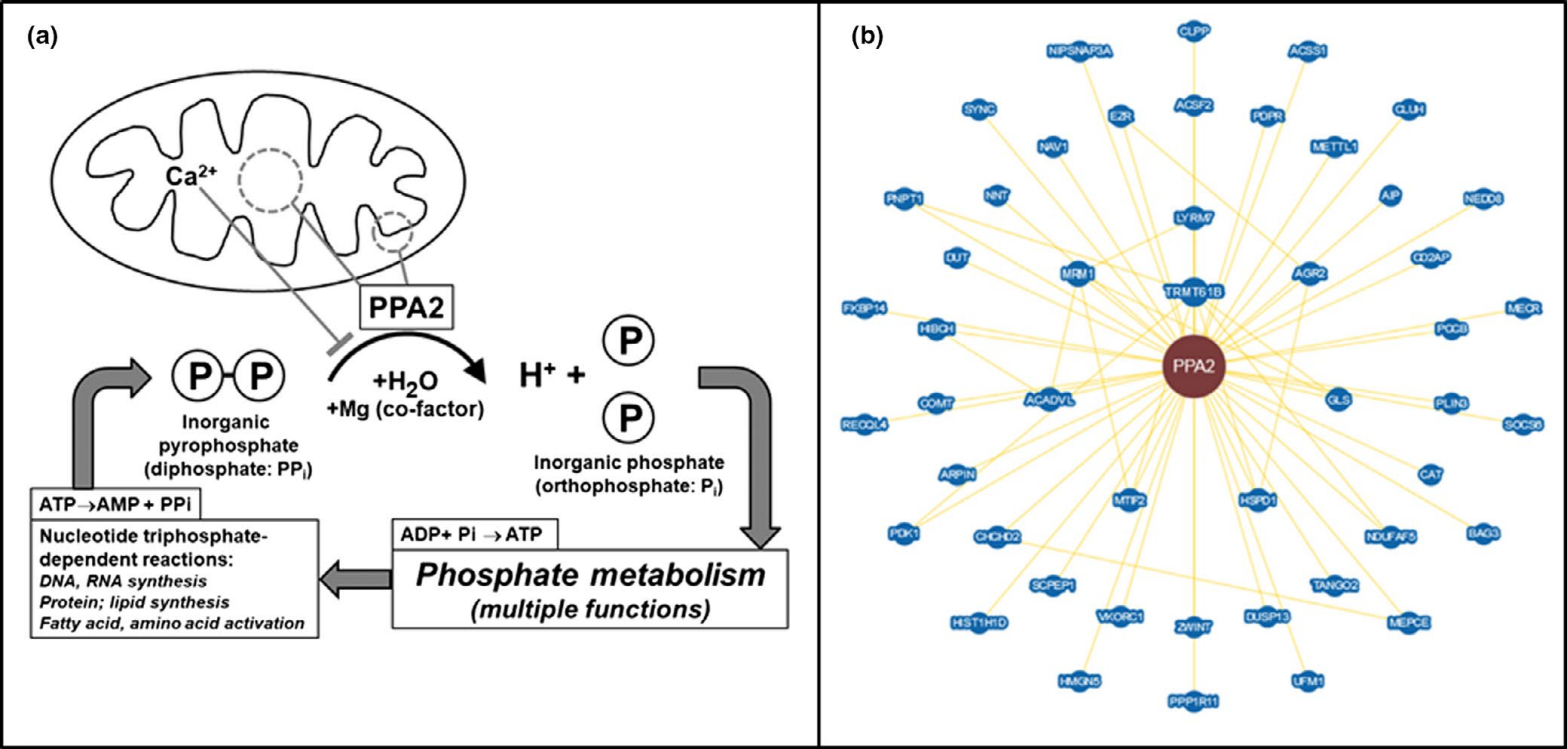
(a) Schematic of PPA2's biological functions, as a pyrophosphatase located in the mitochondrial matrix and inner membrane. Pyrophosphate is generated by nucleotide‐dependent reactions, including DNA and RNA synthesis (including tRNA aminoacylation reactions and cAMP/cGMP synthesis), protein and lipid synthesis, and activation of fatty acids and amino acids. With Mg2+ as a cofactor, PPA2 hydrolyzes inorganic pyrophosphate (PPi) to inorganic phosphate (Pi); this reaction is inhibited by calcium. This reaction energetically favors ongoing nucleotide‐dependent reactions, and inorganic phosphate is used for phosphate metabolism. Biological roles served by PPA2 include the following: mtDNA maintenance; oxidative phosphorylation and generation of ATP; ROS (reactive oxygen species) homeostasis; mitochondrial membrane potential (ΔΨ) regulation; and possibly, communication between mitochondria and nucleus (retrograde signaling pathway). (b) BioGRID showed 49 total interactors (nodes) and 63 interactions (edges), with 48 unique interactors representing diverse mitochondrial and cellular processes (https://thebiogrid.org/117979, accessed 06/30/19)

The yeast homologue of the mitochondrial *PPA2* is required for mitochondrial DNA maintenance and yeast cells lacking the enzyme exhibit mitochondrial DNA depletion. However, a small study of 13 patients with mitochondrial depletion syndromes found no pathogenic mutations identified in the *PPA2* of these patients and therefore, investigators did not believe *PPA2* mutations are a common cause of mitochondrial diseases in humans (Curbo et al., 2006). Three recent studies investigated patients' cells or introduced mutations into yeast as a model organism to gain further mechanistic insights into the biology of *PPA2* (Guimier et al., 2016; Kennedy et al., 2016; Vasilescu et al., 2018; see Table 2). Both predictive modeling and empirical cellular assays demonstrated several variants to be deleterious. Molecular modeling of *PPA2* variants predicted compromised stability of the protein; unfavorable steric, or electrostatic interactions leading to destabilization/misfolding of hPPA2; and/or impairment of enzymatic function due to disruption of hydrogen bonds. Cellular data from various cells and tissues (fibroblasts, heart, skeletal muscle) showed decreases in complex I, complex IV, and possibly decreased mitochondrial number, as well as alterations in FCCP‐stimulated oxygen consumption rates, but even the same variant could yield different results in different patients. Recombinant modeling in *E. coli* suggested that different variants would lead to somewhat different residual protein activities, while PPA2Δ (knockout) yeast exhibited lower antioxidant concentrations, mtDNA depletion, lower oxygen consumption (with reduced complexes III and IV) and reduced ATP synthesis, growth defects, and an inability to maintain the mitochondrial membrane potential. A new study investigating the effects of *PPA2* deletion on yeast longevity also implicated a role in retrograde (mitochondrial‐to‐nucleus) signaling (Muid, Kimyon, Rez, Karakaya, & Koc, 2019). The biological functions of PPA2 are summarized in Figure 1.

**Table 2:** Predicted and empirical abnormal PPA2 protein function(s)

Ref.	Variant	Molecular modeling predictions	Patient tissue(s)	Patient tissue assays
G16	c.182C > T (p.Ser61Phe); c.881A > C (p.Gln294Pro)	The altered residues in family F1 are located near the surface of the protein. All the substitutions are predicted to lead to destabilization/misfolding of hPPA2.	Fibroblasts	Western blotting showed human PPA2 protein steady‐state level is decreased in affected individuals' fibroblasts; SOD4 levels were normal.
	c.280A > G (p.Met94Val); c.514G > A (p.Glu172Lys)	The p.Met94Val and p.Glu172Lys substitutions fall within the pyrophosphatase domain. All the substitutions are predicted to lead to destabilization/misfolding of hPPA2.	Fibroblasts	Western blotting showed hPPA2 protein steady‐state level is decreased in affected individuals' fibroblasts; SOD4 levels were normal.
	c.318G > T (p.Met106Ile); c.514G > A (p.Glu172Lys)	The p.Met106Ile, and p.Glu172Lys substitutions fall within the pyrophosphatase domain. All the substitutions are predicted to lead to destabilization/misfolding of hPPA2.	N/A	N/A
	Functional testing: Yeast model (ppa2Δ)	N/A	Yeast	Inability of yeast to grow on nonfermentable substrates, leading rapidly to cell populations mostly lacking mitochondrial DNA (rho^0^ cells). There was a 60%–80% decrease in oxygen consumption, owing to reduced complexes III and IV with decreased mitochondrial ATP synthesis. Mutants could not maintain an electrical potential across the mitochondrial inner membrane.
K16	c.500C > T (p.Pro167Leu); c.500C > T (p.Pro167Leu)	N/A	Cardiac tissue; fibroblasts; skeletal muscle	Respiratory chain function: Varied from normal to moderate reduction in complex I and IV activities in cardiac tissue and was normal in fibroblasts and skeletal muscle tissue.
	c.500C > T (p.Pro167Leu); c.500C > T (p.Pro167Leu)	N/A	Fibroblasts	Western blotting: Normal amounts of PPA2 protein in fibroblast mitochondria from individuals P5, P6, and P7 but decreased amount in P9. In vitro pyrophosphatase activity: Significantly decreased in isolated fibroblast mitochondria from affected individuals P5, P7, and P9.
	c.500C > T (p.Pro167Leu); c.500C > T (p.Pro167Leu)	N/A	Skeletal muscle	Western blotting: In autopsied muscle of P9, the amount of PPA2 protein was decreased, although it appeared to be normal in P6, who carried the same PPA2 mutation.
	c.500C > T (p.Pro167Leu); c.500C > T (p.Pro167Leu)	N/A	Heart tissue	Western blotting: In the cardiac autopsy sample of P7, PPA2, and complex I subunit levels were decreased as was the expression of the mitochondrial marker proteins, suggestive of a more general reduction of mitochondrial number possibly due to changes in tissue composition.
	c.500C > T (p.Pro167Leu); c.500C > T (p.Pro167Leu)	N/A	Fibroblasts	Seahorse XF data: Basal respiration and oligomycin‐inhibited OCR was similar in affected individuals (P5, P6, and P7) but after the addition of the mitochondrial uncoupler FCCP, affected individuals exhibited a higher activity than controls. The reserve respiratory capacity was twice as high in PPA2‐deficient fibroblasts compared to controls.
	c.500C > T (p.Pro167Leu)	Disruption of the secondary structure of PPA2	N/A	N/A
	c.514G > A (p.Glu172Lys)	Disruption of at least three hydrogen bonds between interacting protein chains near the surface of the enzyme's active site; any disruption of the active site may impair enzymatic function of PPA2	N/A	N/A
	Functional testing: *E. coli* model (BL21(DE3)pLysS strain)	N/A	*E. coli*	Compared to wild‐type, the p.Pro167Leu and p.Glu172Lys variants showed 5%–10% residual activity of normal. The p.Pro228Leu variant had a residual activity of 24%–28% compared to wild‐type.
	Functional testing: Yeast model (ppa2 Δ BY4742)	N/A	Yeast	Growth defect of PPA2 knockout (ppa2 Δ BY4742) yeast was detected on diamide‐containing media, which lowers antioxidant concentrations. The increased diamide sensitivity of PPA2‐deficient yeast therefore suggests reduced levels of antioxidants.
V18	c.514G > A (p.Glu172Lys); c.556G > A (p.Val186Met)	Compromised stability of the protein	Fibroblasts	Reduced complex IV (reduced mtCO1 and especially mtCO2)

Note

“OCR”, oxygen consumption rate. References: “G16” (Guimier et al., 2016); “K16” (Kennedy et al., 2016); “V18” (Vasilescu et al., 2018). *PPA2*: GenBank Reference Sequence NG_053007.1.

Not all patients had cells available for assays.

Despite what we know of the biology of PPA2, admittedly we do not have a true understanding of how the cellular deficits lead to organ‐level dysfunctions that most likely leads to death, cardiomyopathy, lethal arrhythmias, or predeliction to a rapidly fatal myocarditis. Nevertheless, we would expect the numerous interactions of PPA2 with other mitochondrial and cellular proteins (Figure 1b) as curated in BioGRID (Oughtred et al., 2019; Stark et al., 2006) to lead to wide‐ranging effects of PPA2 deficiency.

In summary, we describe two sisters, approximately 1 year of age, who died suddenly and were diagnosed by molecular autopsy to carry biallelic variants in *PPA2*. This gene encodes a mitochondrially located inorganic pyrophosphatase and is implicated in progressive and lethal cardiomyopathies. Our cases add additional details to those reported thus far, and broaden the spectrum of clinical and molecular features of *PPA2* variants.
